# Expression control of the AMPK regulatory subunit and its functional significance in yeast ER stress response

**DOI:** 10.1038/srep46713

**Published:** 2017-04-21

**Authors:** Yuichi Kimura, Kenji Irie, Tomoaki Mizuno

**Affiliations:** 1 Department of Molecular Cell Biology, Faculty of Medicine, University of Tsukuba, Tsukuba, Japan

## Abstract

AMP-activated protein kinase (AMPK) is an evolutionarily conserved heterotrimeric kinase complex consisting of a catalytic subunit, α, and two regulatory subunits, β and γ. Previously, we demonstrated that Snf1, the *Saccharomyces cerevisiae* ortholog of AMPK, negatively regulates the unfolded protein response (UPR) pathway and the Hog1 MAP kinase pathway in ER stress response. However, it remains unclear how the alternate three β subunits, Sip1, Sip2, and Gal83, of the Snf1 complex participate in ER stress response. Here, we show that Gal83 plays a major role in Snf1-mediated downregulation of the UPR and Hog1 pathways. Gal83 is the most abundant β subunit in the normal state and further induced by ER stress. This induction is mediated via activation of the *GAL83* promoter by the UPR. When expressed under the control of the *GAL83* promoter, Sip2 exhibits potent functional activity equivalent to Gal83. Our results suggest that the functional significance of the β subunit of Snf1 AMPK in ER stress response is defined by modulation of the expression level through regulation of the promoter activity.

Newly synthesized secretory or membrane proteins are folded and glycosylated in the endoplasmic reticulum (ER). Perturbation of ER homeostasis caused by environmental or developmental changes results in an accumulation of aberrant proteins within the ER. This condition is designated as ER stress. When ER stress is sensed, cells initiate adaptive responses to alleviate ER stress^1^,^2^. In *Saccharomyces cerevisiae*, the unfolded protein response (UPR) signaling pathway composed of Ire1 and Hac1 plays a central role in ER stress response^1^,^2^. Ire1 is an ER transmembrane protein acting as a sensor of aberrant proteins. Ire1 becomes activated in response to ER stress and then excises the translation-inhibitory intron from *HAC1* mRNA. Spliced *HAC1* mRNA produces a transcriptional activator, which consequently induces expression of target genes. The gene expression program activated by Hac1 increases ER-resident chaperones and proteins functioning ER-associated degradation, thus alleviating ER stress. In addition to the UPR, the stress responsive MAP kinases, such as Mpk1 and Hog1, become activated by ER stress and function to protect yeast cells from ER stress^3^,^4^,^5^,^6^.

AMP-activated protein kinase (AMPK) acts as a key sensor of cellular energy status in eukaryotic cells^7^,^8^,^9^. The budding yeast ortholog of AMPK, Snf1, not only plays an essential role in the response to glucose deprivation, but also controls adaptive responses to a variety of environmental stresses, such as oxidative and heat stresses^7^,^10^. Similar to mammalian AMPK, Snf1 forms a heterotrimeric complex with two regulatory subunits, β and γ. The γ subunit is encoded by the *SNF4* gene^7^. On the other hand, the β subunits are encoded by three genes, *SIP1, SIP2*, and *GAL83*, and one of them is utilized in each complex^7^,^11^,^12^,^13^. Although these β subunits share overlapping functions, they also display distinctive features^14^. For instance, their carboxyl-terminal sequences are conserved and mediate their interaction with Snf1 and Snf4; however, they have divergent amino-terminal sequences that direct the distinct subcellular localization of the Snf1 complex^15^. Previous studies have also demonstrated that the β subunits specify substrate preferences and stress response capacities of the Snf1 complex^7^,^16^,^17^. Furthermore, it has been reported that the expression levels of the β subunits significantly differ from each other^15^.

Previous studies from us and another group demonstrated the involvement of Snf1 in ER stress response^18^,^19^. We have revealed Snf1 as a negative regulator of the UPR pathway and the Hog1 MAPK pathway in ER stress response^19^. The deletion of the *SNF1* gene caused increased resistance to ER stress. The cells lacking all three β subunits displayed ER stress tolerance indistinguishable from that observed in the *snf1* mutants. However, it has remained unclear which β subunit is important for the negative regulation of the UPR and Hog1 pathways. In this study, we found that Gal83 plays a major role in Snf1-mediated downregulation of the UPR and Hog1 during ER stress response. Among the β subunits, Gal83 is the most abundant under normal conditions, and its expression is further induced by ER stress in a manner dependent on the UPR. When *SIP2* was expressed from the *GAL83* promoter, loss of Gal83 could be effectively complemented. These results suggest that the functional significance of Gal83 as the AMPK β subunit in ER stress response is defined by its promoter.

## Results

### Gal83 is the principal β subunit of the Snf1 complex in ER stress response

**Figure 1:**
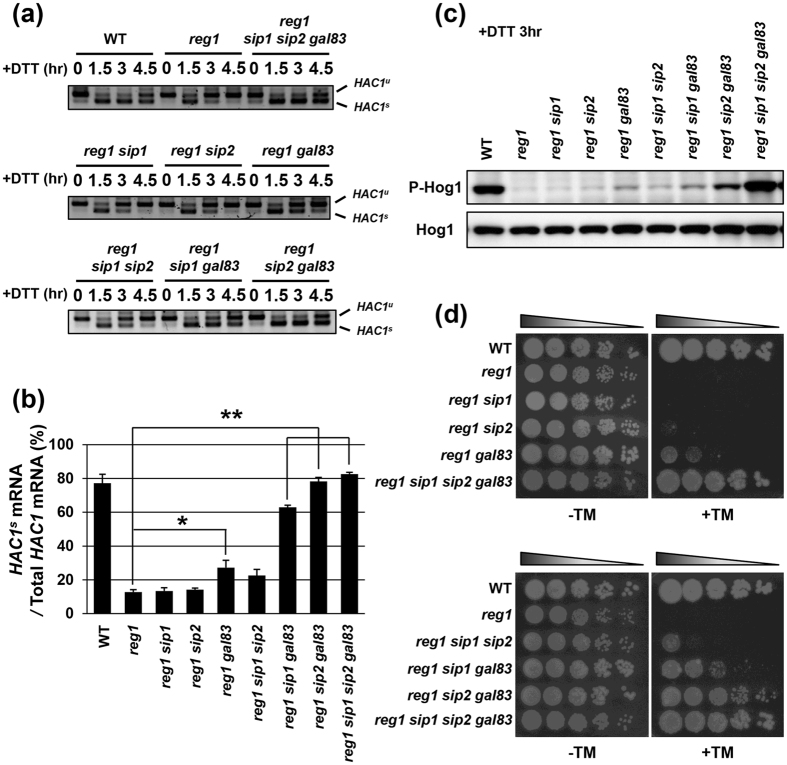
Gal83 acts as a major β subunit in regulation of the UPR and Hog1. (**a**,**b**) Splicing of *HAC1* mRNA after DTT treatment. Wild-type (WT) and indicated mutant strains were grown at 25 °C until exponential phase and treated with 4 mM dithiothreitol (DTT) for the indicated time. Total RNAs prepared from each strain were subjected to RT-PCR of *HAC1*. Positions of unspliced *HAC1 (HAC1*^*u*^) and spliced *HAC1 (HAC1*^*s*^) are indicated. The mean of *HAC1*^*s*^/(*HAC1*^*u*^ + *HAC1*^*s*^) at 3 hours after DTT addition with SEM (n = 4) is shown in (**b**). *P < 0.05 and **P < 0.01 as determined by Tukey’s test. Original data are presented in Supplementary Fig. 4. (**c**) Hog1 activation after DTT treatment. Wild-type (WT) and indicated mutant strains were grown at 25 °C until exponential phase and treated with 4 mM dithiothreitol (DTT) for 3 hours. Extracts prepared from each cell were immunoblotted with anti-phospho-p38 (P-Hog1) and anti-Hog1 antibodies. Original data are presented in Supplementary Fig. 5. (**d**) Loss of Gal83 has the strongest influence on ER stress sensitivity. Wild-type (WT) and indicated mutant strains were spotted onto YPD medium lacking or containing 0.5 μg/ml tunicamycin (TM) and incubated at 25 °C.

To investigate which β subunits are involved in regulation of the UPR and Hog1 pathways, we employed *reg1* mutation which causes Snf1 hyperactivation. The kinase activity of Snf1 is regulated through phosphorylation of Thr-210 located in its kinase domain^20^,^21^: Snf1 is phosphorylated and activated by three upstream kinases, Sak1, Tos3, and Elm1^22^,^23^,^24^; Snf1 inactivation is mediated by the Reg1-Glc7 protein phosphatase 1 complex^25^,^26^. Reg1 is the regulatory subunit that guides Glc7 catalytic subunit toward Snf1^27^,^28^. Previously, we showed that Snf1 hyperactivation caused by *reg1* mutation leads to rapid downregulation of the UPR activity^19^. The reverse transcription-PCR (RT-PCR) analysis revealed that a large fraction of *HAC1* mRNA remained the unspliced form (*HAC1*^*u*^) in wild-type cells under unstressed conditions (Fig. 1a). Exposure to dithiothreitol (DTT), which causes ER stress by blocking disulfide bond formation in the ER, induced *HAC1* mRNA splicing. The amount of the spliced form of *HAC1* mRNA (*HAC1*^*s*^) peaked 1.5 to 3 hr after DTT addition and gradually decreased thereafter (Fig. 1a). In *reg1* mutant cells, promotion of *HAC1* mRNA splicing by DTT treatment was apparently normal; however, *HAC1*^*s*^ was decreased rapidly within 3 hr of DTT addition (Fig. 1a). This *reg1* defect could be significantly restored by *snf1* mutation, while cells harboring *snf1* single mutation only exhibited a mild defect in *HAC1* mRNA splicing under unstressed conditions^19^. Thus, alteration of the kinetics of *HAC1* mRNA splicing caused by *reg1* mutation was expected to be highly sensitive to reduction of Snf1 function. Indeed, loss of all three β subunits clearly suppressed rapid downregulation of *HAC1* mRNA splicing observed in *reg1* mutant cells (Fig. 1a and b). To further explore the role of the β subunits in the UPR regulation, we examined *HAC1* mRNA splicing in single and double mutant cells of the β subunits in a *reg1* mutant background (Fig. 1a and b). The *gal83* mutation slightly delayed downregulation of *HAC1* mRNA splicing, but neither *sip1* nor *sip2* mutation did. The *gal83 sip1* and *gal83 sip2* double mutations significantly delayed downregulation of *HAC1* mRNA splicing, while *sip1 sip2* double mutation did not. These results suggest that Gal83 is the most important β subunit for Snf1-mediated regulation of the UPR activation in response to DTT. To test whether similar effects were observed using different types of the ER stressor, we next monitored *HAC1* mRNA splicing in cells exposed to tunicamycin, which causes ER stress by inhibition of N-linked glycosylation (Supplementary Fig. 1a and b). In wild-type cells, the high amount of *HAC1*^*s*^ was kept over 7.5 hr of tunicamycin addition. In contrast, in *reg1* mutant cells, a gradual decrease in *HAC1*^*s*^ was detected within 7.5 hr after tunicamycin addition. Thus, tunicamycin-induced activation of the UPR was sustained long-term compared to DTT; however, *reg1* mutation caused a significant decline in the UPR activity, similar to the case when cells were treated with DTT. Furthermore, this *reg1* phenotype could be suppressed by *gal83 sip1* and *gal83 sip2* double mutations, but not by *sip1 sip2* double mutation. Taken together, these results suggest that Gal83 acts as the main β subunit of the Snf1 complex in the UPR regulation.

We previously showed that Snf1 is involved in negative regulation of the UPR pathway by using W303 derivative strains^19^. To investigate whether the inhibitory effect of the Snf1 complex on the UPR is restricted to the W303 background, we employed BY4741 derivatives as budding yeast cells harboring a different genetic background. We first compared the kinetics of *HAC1* mRNA splicing between wild-type and *reg1* mutant cells, and found that an accelerated decline in the UPR activity was occurred in *reg1* mutant cells (Supplementary Fig. 2a and b). Next, we tested double mutant cells of the β subunits in a *reg1* mutant background. The *gal83 sip1* and *gal83 sip2* double mutations modestly delayed downregulation of *HAC1* mRNA splicing, while *sip1 sip2* double mutation did not (Supplementary Fig. 2a and b). Thus, both in the W303 and BY4741 backgrounds, *reg1* mutation downregulates the UPR activity, and this *reg1* defect could be restored by loss of Gal83 in combination with Sip1 or Sip2. Therefore, it is suggested that the mechanism by which Snf1 negatively regulates the UPR is widely used in budding yeast cells with different genetic backgrounds. We hereafter mainly described the results from experiments using W303 derivatives, although similar results were obtained from all experiments we have tried using BY4741 derivatives and they were shown in Supplementary Fig. 2.

Next, we examined which β subunits are involved in regulation of the Hog1 pathway. To monitor Hog1 activity, we used anti-phospho-p38 antibodies that recognize the phosphorylated form of mammalian p38 MAPK. As shown previously^19^, western blot analysis with anti-phospho-p38 antibodies strongly detected the activated Hog1 in wild-type cells treated with DTT (Fig. 1c). However, activated Hog1 level was significantly decreased in *reg1* mutant cells (Fig. 1c). We have also demonstrated that *snf1* mutation could completely restore the reduction of Hog1 activity caused by *reg1* mutation^19^. To investigate the role of the β subunits in regulation of Hog1 activity, we first detected activated Hog1 in the *reg1 sip1 sip2 gal83* quadruple mutant cells. Loss of all three β subunits clearly suppressed the reduction of Hog1 activity caused by *reg1* mutation (Fig. 1c). Therefore, we compared the effects of *sip1, sip2* and *gal83* mutations on *reg1*-caused reduction of Hog1 activity (Fig. 1c). The *gal83* mutation slightly restored Hog1 activity. On the other hand, neither *sip1* nor *sip2* single mutation had an obvious effect on Hog1 activity. We also found that the activated Hog1 levels were increased in the following order: *sip1 sip2* <*sip1 gal83* <*sip2 gal83* <*sip1 sip2 gal83*. Similar observations were seen when cells were exposed to tunicamycin (Supplementary Fig. 1c). These results suggest that Gal83 is the most important β subunit for Snf1-mediated regulation of the Hog1 pathway.

We previously found that *reg1* mutant cells exhibit hypersensitivity to ER stress and this *reg1* phenotype is completely suppressed by *snf1* mutation^19^. To ask whether Gal83 is actually important for regulation of ER stress response, we examined growth of yeast cells on medium containing tunicamycin (Fig. 1d). Similar to *snf1* mutation, loss of all three β subunits suppressed the ER stress sensitive phenotype observed in *reg1* mutant cells. The *reg1* hypersensitivity to ER stress was partially suppressed by *gal83* mutation and to a much lesser extent by *sip2* mutation. In contrast, *sip1* single mutation failed to suppress the ER stress sensitive phenotype of *reg1* mutants. ER stress tolerance caused by *gal83* mutation was significantly enhanced by *sip2* mutation and to a lesser extent by *sip1* mutation. Thus, Gal83 has the strongest influence on ER stress sensitivity among three β subunits. This finding is consistent with the results showing that Gal83 acts as the major β subunit for Snf1-mediated regulation of the UPR and Hog1 pathways.

### The relative functional significance of the β subunits is modulated by Snf1 activation level

**Figure 2:**
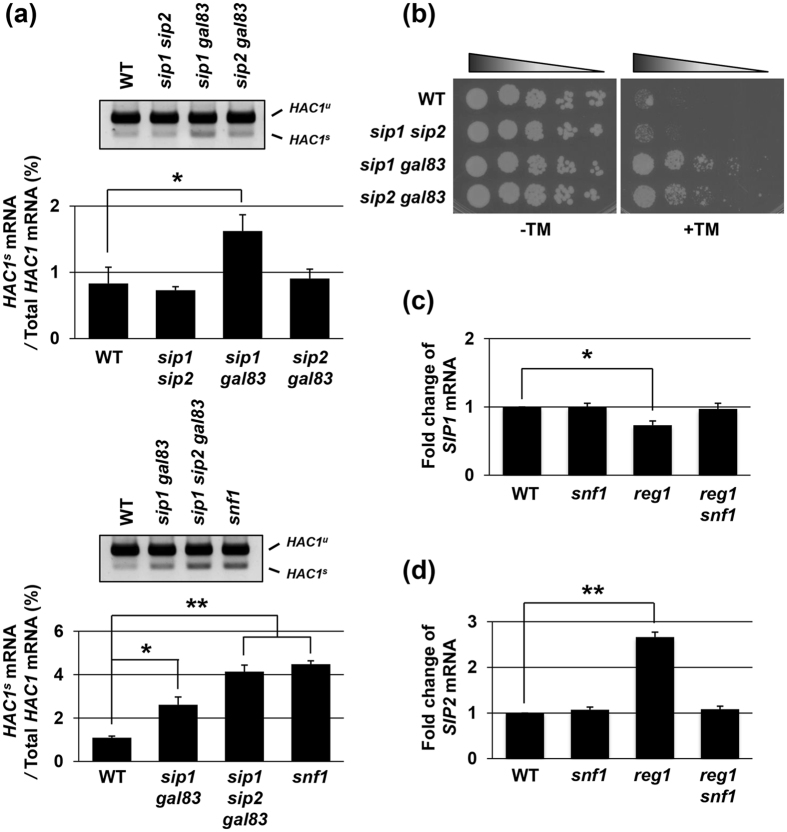
Functional significance of Sip1 and Sip2 is altered by Snf1 activity. (**a**) Splicing of *HAC1* mRNA in the cells lacking two β subunits. Wild-type (WT) and indicated mutant strains were grown at 25 °C until exponential phase, and total RNAs prepared from each strain were subjected to RT-PCR of *HAC1*. Positions of unspliced *HAC1 (HAC1*^*u*^) and spliced *HAC1 (HAC1*^*s*^) are indicated. The data show the mean of *HAC1*^*s*^/(*HAC1*^*u*^ + *HAC1*^*s*^) with SEM (n = 3). *P < 0.05 and **P < 0.01 as determined by Tukey’s test. Original data are presented in Supplementary Fig. 6. (**b**) ER stress sensitivity of the cells lacking two β subunits. Wild-type (WT) and indicated mutant strains were spotted onto YPD medium lacking or containing 1.5 μg/ml tunicamycin (TM) and incubated at 25 °C. (**c**,**d**) The mRNA levels of *SIP1* (**c**) and *SIP2* (**d**). Wild-type (WT) and indicated mutant strains were grown at 25 °C until exponential phase, and total RNAs were prepared from each strain. The mRNA levels were quantified by qRT-PCR analysis, and relative mRNA levels were calculated using *ACT1* mRNA. The data show mean ± SEM (n = 4). *P < 0.05 and **P < 0.01 as determined by Tukey’s test.

Next, we investigated whether the relative contribution of the Snf1 β subunits to the UPR activity seen in the *reg1* mutants could be observed in cells harboring the wild-type *REG1* gene. Previously, we found that *snf1* mutation elevates *HAC1* mRNA splicing under unstressed conditions, but does not cause a significant change in its kinetics during ER stress response^19^. Therefore, we compared *HAC1* mRNA splicing in double mutant cells of the β subunits under unstressed conditions (Fig. 2a). Intriguingly, the *gal83 sip1* double mutation increased the level of *HAC1*^*s*^, while *sip1 sip2* and *gal83 sip2* double mutations did not. We also found that the level of *HAC1*^*s*^ in the *gal83 sip1 sip2* triple mutant cells was higher than that in the *gal83 sip1* double mutant cells. These results suggest that Gal83 and Sip1 have greater potential to regulate the UPR than Sip2 in wild-type backgrounds, and that the relative contribution of three β subunits is slightly different between wild-type and *reg1* mutant backgrounds.

Previously, we showed that the *sip1 sip2 gal83* triple mutant cells were resistant to tunicamycin, although none of their single mutants exhibited the obvious tunicamycin-resistant phenotype^19^. To further clarify involvement of Snf1 β subunits in ER stress response, we tested *sip1 sip2, gal83 sip1* and *gal83 sip2* double mutants for growth on medium containing tunicamycin (Fig. 2b). The *gal83 sip1* and *gal83 sip2* double mutants exhibited the tunicamycin-resistant phenotype, while *sip1 sip2* double mutants did not. This indicates that Gal83 plays a major role in Snf1-mediated ER stress response. Furthermore, *gal83 sip1* mutant cells were more resistant to tunicamycin than *gal83 sip2* mutant cells. This observation suggests that, in contrast to a *reg1* mutant background, Sip1 is more important for the function in Snf1-mediated ER stress response than Sip2 in a wild-type background.

In both wild-type and *reg1* mutant backgrounds, Gal83 acts as the most important β subunit of the Snf1 complex in ER stress response. However, the degree to which Sip1 and Sip2 participate in ER stress response seemed to vary between wild-type and *reg1* mutant backgrounds: Sip1 is more and less important than Sip2 in wild-type and *reg1* mutant backgrounds, respectively. To elucidate the mechanism by which functional significance of Sip1 and Sip2 is altered, we compared the mRNA levels of *SIP1* and *SIP2* in wild-type and *reg1* mutant cells by a quantitative real-time RT-PCR (qRT-PCR). The *SIP1* mRNA level was only modestly reduced by *reg1* mutation (Fig. 2c); in contrast, the *SIP2* mRNA level was significantly increased by *reg1* mutation (Fig. 2d). Furthermore, the increase in *SIP2* mRNA caused by *reg1* mutation was clearly inhibited by loss of Snf1 (Fig. 2d). These results suggest that expression changes of *SIP1* and *SIP2* mRNAs caused by *reg1* mutation-mediated Snf1 activation contribute to enhance the relative functional significance of Sip2 in *reg1* mutant cells.

### Gal83 expression is higher than those of Sip1 and Sip2, and induced by ER stress

**Figure 3:**
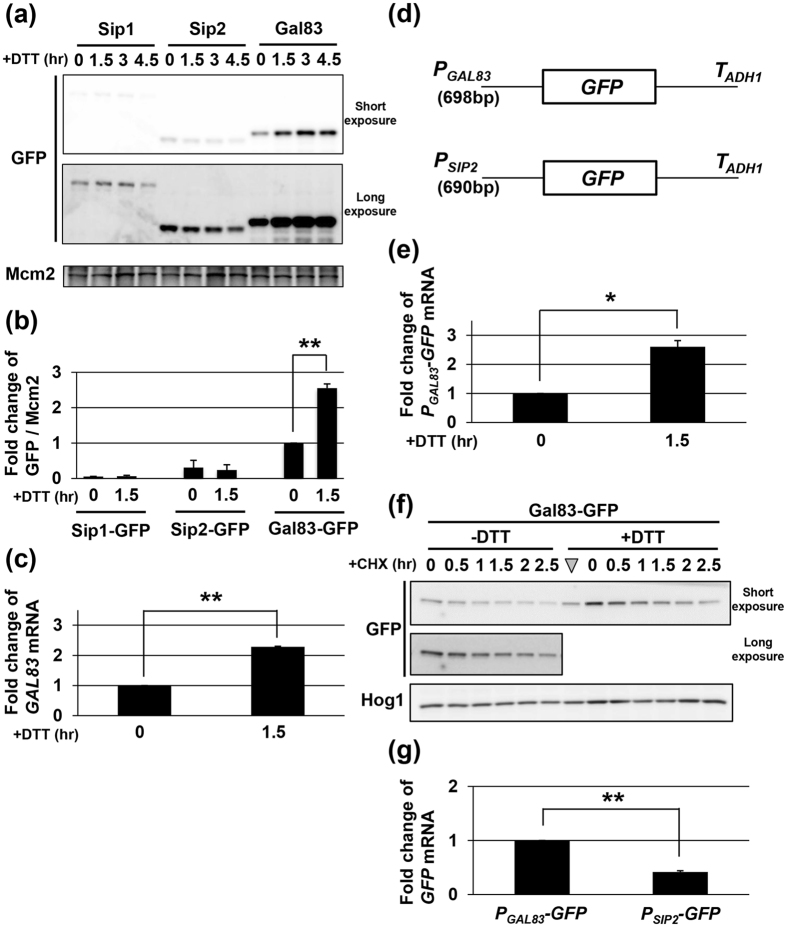
Gal83 is the most abundant β subunit and its expression is further induced by ER stress. (**a**,**b**) The expression levels of Sip1, Sip2 and Gal83 after DTT treatment. Wild-type (WT) strains harboring GFP-tagged *SIP1, SIP2*, or *GAL83* were grown at 25 °C until exponential phase and treated with 4 mM dithiothreitol (DTT) for the indicated time. Extracts prepared from each cell were immunoblotted with anti-GFP and anti-Mcm2 antibodies. The intensities of Sip1-GFP, Sip2-GFP and Gal83-GFP were measured and normalized to Mcm2 level. The values are plotted as the fold change from Gal83-GFP in cells at the time of DTT addition. The data show mean ± SEM (n = 3). **P < 0.01 as determined by Student’s *t*-test. Original data are presented in Supplementary Fig. 7. (**c**) The mRNA levels of *GAL83* after DTT treatment. Wild-type (WT) strains were grown at 25 °C until exponential phase and treated with 4 mM dithiothreitol (DTT) for the indicated time. The mRNA levels were quantified by qRT-PCR analysis, and relative mRNA levels were calculated using *ACT1* mRNA. The data show mean ± SEM (n = 3). **P < 0.01 as determined by Student’s *t*-test. (**d**) Schematic representation of the structure of *P*_*GAL83*_*-GFP* and *P*_*SIP2*_*-GFP*. (**e**) Effects of DTT treatment on expression of *P*_*GAL83*_*-GFP* reporter. Wild-type (WT) cells harboring the integration which expresses GFP under the control of *GAL83* promoter were grown at 25 °C until exponential phase and treated with 4 mM dithiothreitol (DTT) for the indicated time. The *GFP* mRNA levels were quantified by qRT-PCR analysis, and relative mRNA levels were calculated using *ACT1* mRNA. The data show mean ± SEM (n = 3). *P < 0.05 as determined by Student’s *t*-test. (**f**) Stability of Gal83. Cells expressing Gal83-GFP were treated with or without 4 mM dithiothreitol (DTT) for 1.5 hours, and then treated with 0.2 mg/ml cycloheximide (CHX) for the indicated time. The triangle indicates the point when DTT was added. Extracts prepared from each cell were immunoblotted with anti-GFP and anti-Hog1 antibodies. Hog1 was used as a loading control, since it has been reported to be a stable protein^35^. (**g**) Comparison between *GFP* expression levels from *P*_*GAL83*_*-GFP* and *P*_*SIP2*_*-GFP* reporters. Wild-type (WT) cells harboring the integration which expresses GFP under the control of *GAL83* or *SIP2* promoters were grown at 25 °C until exponential phase. The *GFP* mRNA levels were quantified by qRT-PCR analysis, and relative mRNA levels were calculated using *ACT1* mRNA. The data show mean ± SEM (n = 3). **P < 0.01 as determined by Student’s *t*-test.

**Figure 4:**
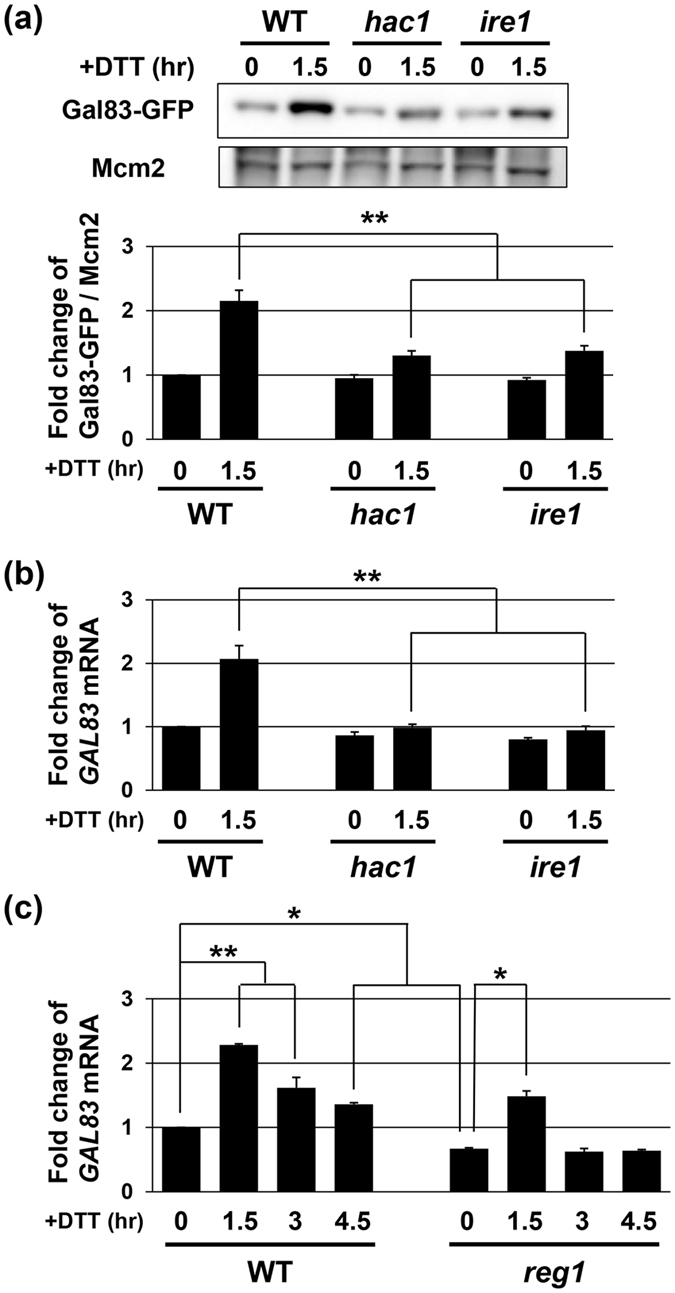
Gal83 expression is positively regulated by the UPR. (**a**) Effects of the *hac1* and *ire1* mutations on DTT-induced upregulation of Gal83. Wild-type (WT) and *hac1* and *ire1* mutant strains harboring GFP-tagged *GAL83* were grown at 25 °C until exponential phase and treated with 4 mM dithiothreitol (DTT) for the indicated time. Extracts prepared from each cell were immunoblotted with anti-GFP and anti-Mcm2 antibodies. The intensities of Gal83-GFP were measured and normalized to Mcm2 level. The values are plotted as the fold change from wild-type cells at the time of DTT addition. The data show mean ± SEM (n = 3). **P < 0.01 as determined by Tukey’s test. Original data are presented in Supplementary Fig. 8. (**b**) Effects of the *hac1* and *ire1* mutations on DTT-induced upregulation of *GAL83* mRNA. Wild-type (WT) and *hac1* and *ire1* mutant strains were grown at 25 °C until exponential phase and treated with 4 mM dithiothreitol (DTT) for the indicated time. The mRNA levels were quantified by qRT-PCR analysis, and relative mRNA levels were calculated using *ACT1* mRNA. The data show mean ± SEM (n = 3). **P < 0.01 as determined by Tukey’s test. (**c**) Effects of the *reg1* mutation on ER stress-induced upregulation of *GAL83* mRNA. Wild-type (WT) and *reg1* mutant strains were grown at 25 °C until exponential phase and treated with 4 mM dithiothreitol (DTT) for the indicated time. The mRNA levels were quantified by qRT-PCR analysis, and relative mRNA levels were calculated using *ACT1* mRNA. The data show mean ± SEM (n = 3). *P < 0.05 and **P < 0.01 as determined by Tukey’s test.

In order to elucidate why Gal83 is the most important for Snf1 to negatively regulate ER stress response, we compared the expression levels of Sip1, Sip2, and Gal83. We generated yeast strains carrying the carboxyl-terminally GFP-tagged genes and quantitated their expression by western blot analysis with anti-GFP antibodies (Fig. 3a and b). In the normal state, the protein abundance of Gal83 was higher than those of Sip1 and Sip2. This observation is consistent with the previous report^17^. Intriguingly, Gal83, but neither Sip1 nor Sip2, was increased following exposure to DTT (Fig. 3a and b). Induction of Gal83 was also observed when cells were treated with tunicamycin (Supplementary Fig. 1d). To investigate how the expression level of Gal83 is upregulated by ER stress, we quantitated *GAL83* mRNA by qRT-PCR. We found that the *GAL83* mRNA level is transiently increased by ER stress: the amount of *GAL83* mRNA peaked 1.5 hr after DTT treatment and decreased thereafter (Figs 3c and 4c). Similar induction was observed when cells were exposed to tunicamycin and in strains harboring the BY4741 background (Supplementary Figs 1e and 2c). Next, we examined whether increased expression of *GAL83* is due to its transcriptional activation by ER stress. To address this, we generated a *P*_*GAL83*_*-GFP* reporter, consisting of the 5′ upstream region of the *GAL83* gene to drive GFP expression (Fig. 3d), and monitored the amount of *GFP* mRNA by qRT-PCR. *GFP* expression from the *P*_*GAL83*_*-GFP* reporter was increased after treatment with DTT and tunicamycin (Fig. 3e and Supplementary Fig. 1f), suggesting that the *GAL83* promoter is activated by ER stress. We also tested the possibility that Gal83 is stabilized by ER stress, which consequently contributes to upregulation of the protein level of Gal83. Cells expressing Gal83-GFP were treated with or without DTT, and the protein level of Gal83-GFP was examined following cycloheximide treatment. However, the stability of Gal83-GFP was apparently unaffected by DTT (Fig. 3f). Based on the findings that the induction level of the *P*_*GAL83*_*-GFP* reporter was comparable with that of Gal83-GFP protein (Fig. 3b and e), Gal83 expression is induced by ER stress through transcriptional activation of the *GAL83* gene.

We next attempted to identify the regulator of *GAL83* expression. Previous studies demonstrated that in budding yeast, several signaling pathways, including the UPR, Mpk1, Hog1, and Snf1, become activated in response to ER stress^1^,^2^,^3^,^4^,^5^,^6^,^19^. Under our experimental conditions, the UPR pathway consisting of Ire1 and Hac1 was quickly activated after DTT addition and thereafter downregulated (Fig. 1a and b). On the other hand, our previous analyses revealed that activation of Hog1 and Snf1 was occurred comparatively late after exposure to DTT and maintained long-term^19^. Furthermore, the activation time course of Mpk1 was similar to those of Hog1 and Snf1 under our experimental conditions (Supplementary Fig. 3). Therefore, we examined whether the UPR pathway is involved in transcriptional activation of the *GAL83* gene. We found that induction of Gal83 protein and *GAL83* mRNA following exposure to DTT was impaired in *hac1* and *ire1* mutant cells (Fig. 4a and b). The *hac1* and *ire1* mutations inhibited induction of *GAL83* mRNA when cells were exposed to tunicamycin and in strains harboring the BY4741 background (Supplementary Figs 1e and 2c). These results indicate that the UPR pathway induces *GAL83* expression during ER stress response.

The *reg1* mutation leads to Snf1 hyperactivation and consequent decreased activity of the UPR pathway during ER stress response^19^ (Fig. 1a). This observation raised the possibility that *GAL83* expression level was reduced by *reg1* mutation. To test this possibility, we measured the amount of *GAL83* mRNA in *reg1* mutant cells. We found that *GAL83* mRNA levels were reduced by *reg1* mutation (Fig. 4c). Similar result was obtained in *reg1* mutant cells harboring the BY4741 background (Supplementary Fig. 2d). These results suggest that in ER stress response, Snf1 downregulates expression of its regulatory subunit by inhibiting the UPR activity.

### Sip2 expressed from the *GAL83* promoter compensates for loss of Gal83

**Figure 5:**
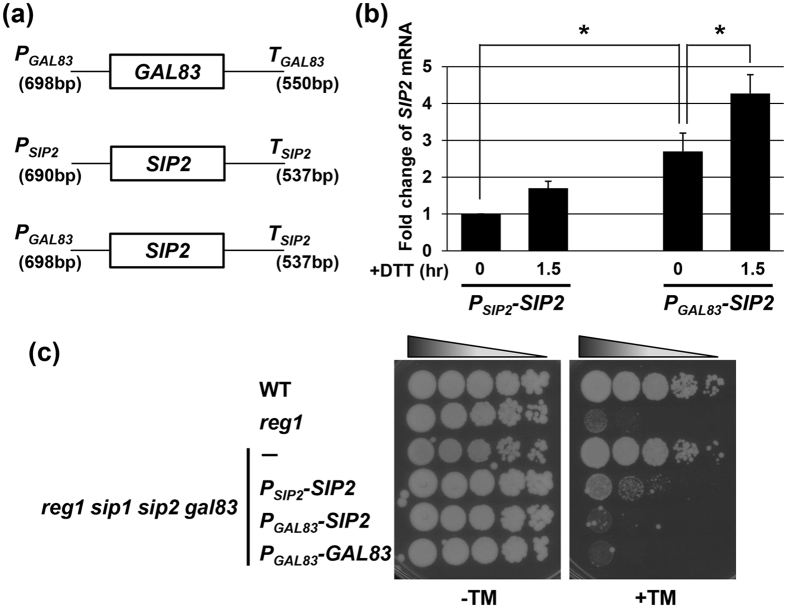
Upregulation of Sip2 expression compensates for loss of Gal83. (**a**) Schematic representation of the structure of the *PGAL83-GAL83, PSIP2-SIP2* and *P*_*GAL83*_*-SIP2* integrations. (**b**) Comparison between *SIP2* expression levels from *PSIP2-SIP2* and *P*_*GAL83*_*-SIP2*. The *reg1 sip1 sip2 gal83* quadruple mutant strains harboring the integration of *P*_*SIP2*_*-SIP2* or *P*_*GAL83*_*-SIP2* were treated with 4 mM dithiothreitol (DTT) for the indicated time. The *SIP2* mRNA levels were quantified by qRT-PCR analysis, and relative mRNA levels were calculated using *ACT1* mRNA. The data show mean ± SEM (n = 3). *P < 0.05 as determined by Tukey’s test. (**c**) Effects of the *P*_*GAL83*_*-GAL83, P*_*SIP2*_*-SIP2* and *P*_*GAL83*_*-SIP2* integrations on ER stress sensitivity. Wild-type (WT) and *reg1* single and *reg1 sip1 sip2 gal83* quadruple mutant strains harboring the integration of *P*_*GAL83*_*-GAL83, P*_*SIP2*_*-SIP2*, or *P*_*GAL83*_*-SIP2* were spotted onto YPD medium lacking or containing 0.5 μg/ml tunicamycin (TM) and incubated at 25 °C.

Comparison of the expression levels among the β subunits led us to hypothesize that their protein abundance, but not their protein structure, determines their demands for ER stress response mediated by Snf1. To test this hypothesis, we first generated a *P*_*SIP2*_*-GFP* reporter, consisting of the 5′ upstream region of the *SIP2* gene to drive GFP expression, and compared its activity to express *GFP* with a *P*_*GAL83*_*-GFP* reporter. The qRT-PCR analysis showed that, under unstressed conditions, *GFP* mRNA level from a *P*_*SIP2*_*-GFP* reporter was half of that from a *P*_*GAL83*_*-GFP* reporter (Fig. 3g). Thus, the difference in promoter activity was reflected in their protein level (Fig. 3b and g). We next generated a *P*_*GAL83*_*-SIP2* construct, which expresses *SIP2* under the control of the *GAL83* promoter (Fig. 5a). To confirm that the expression pattern of *SIP2* mRNA from the *P*_*GAL83*_*-SIP2* integration mimics that of *GAL83* mRNA, we quantified *SIP2* mRNA in the quadruple mutant cells harboring the wild-type *SIP2 (P*_*SIP2*_*-SIP2*) or *P*_*GAL83*_*-SIP2* integration by qRT-PCR. As expected, we found that under unstressed conditions, *SIP2* mRNA was expressed at a higher level from the *P*_*GAL83*_*-SIP2* integration than from the *P*_*SIP2*_*-SIP2* integration, and that expression of *SIP2* mRNA from the *P*_*GAL83*_*-SIP2* integration was increased by DTT and tunicamycin (Fig. 5b and Supplementary Fig. 1g). Then, we compared the ability of *P*_*GAL83*_*-SIP2* to alter the phenotype caused by *reg1 sip1 sip2 gal83* quadruple mutation with those of *P*_*GAL83*_*-GAL83* and *P*_*SIP2*_*-SIP2*. The *reg1 sip1 sip2 gal83* quadruple mutant cells were resistant to ER stress, while the *reg1* single mutant cells were sensitive to ER stress (Fig. 5c). When harboring the *P*_*GAL83*_*-GAL83* integration, the *reg1 sip1 sip2 gal83* quadruple mutants exhibited ER stress hypersensitivity, similar to the *reg1* single mutants. However, the *reg1 sip1 sip2 gal83* quadruple mutants harboring the *P*_*SIP2*_*-SIP2* integration displayed the intermediate phenotype between the *reg1 sip1 sip2 gal83* quadruple mutant and the *reg1* single mutant. These results indicate that the *P*_*GAL83*_*-GAL83* integration has a stronger activity to complement *sip1 sip2 gal83* triple mutations than the *P*_*SIP2*_*-SIP2* integration. The activity of the *P*_*GAL83*_*-SIP2* integration was the same as that of the *PGAL83-GAL83* integration. This result suggests that in ER stress response, the difference in the promoter activity between the *GAL83* and *SIP2* genes is more important than their difference in the protein structure.

## Discussion

In budding yeast, the UPR signaling pathway, composed of Ire1 ER transmembrane sensor and Hac1 transcription factor, plays a pivotal role in ER stress response^1^,^2^. We previously demonstrated that the budding yeast ortholog of AMPK, Snf1, acts as a negative regulator of the UPR^19^. Snf1 is also involved in downregulation of the Hog1 MAPK during ER stress response. Similar to mammalian AMPK, Snf1 forms a heterotrimeric complex with two regulatory subunits, β and γ^7^. Budding yeast expresses three β subunits, and one of them is incorporated into each Snf1 complex^7^. However, it has remained unclear which β subunit functions in ER stress response mediated by Snf1. Here, we revealed that Gal83 makes the greatest contribution to the regulation of UPR and Hog1 among three β subunits. Consistently, loss of Gal83 caused stronger resistance to ER stress than those of Sip1 or Sip2. These indicate that Gal83 is the principal β subunit in ER stress response.

Our analyses utilizing a highly sensitive *reg1* mutant background showed the relative contribution of three β subunits to the UPR regulation is identical to that to Hog1 regulation. This implies that the UPR and Hog1 pathways are regulated through a similar mechanism involving Snf1. However, the demands of Snf1 activity for the UPR and Hog1 pathways are likely to differ from each other. The decreased *HAC1* mRNA splicing caused by *reg1* mutation could be suppressed by either *sip1 gal83* or *sip2 gal83* double mutations at the level similar to the *sip1 sip2 gal83* triple mutations. In contrast, *sip1 gal83* and *sip2 gal83* double mutations lead to weak suppression of decreased Hog1 activity caused by *reg1* mutation, compared to the *sip1 sip2 gal83* triple mutations. Therefore, it is suggested that Snf1-mediated regulation of the UPR and Hog1 pathways requires the relatively high and low activities of Snf1, respectively.

Previous studies have characterized the various functional differences of the β subunits^7^,^14^,^15^,^16^,^17^. For instance, only Sip2 has been implicated in intrinsic aging^29^. However, there appear to be little functional differences in ER stress response, as upregulation of Sip2 expression level using *GAL83* promoter could effectively complement loss of Gal83. Rather, their abundance controlled by the promoter activity may be critical to define their importance in ER stress response. Consistent with previous studies^15^, we observed that Gal83 is the most abundant in normal conditions among three β subunits. Thus, the greatest contribution of Gal83 to Snf1-mediated ER stress response is consistent with the highest expression level of Gal83. However, there may be the difference in activity between Sip1 and Sip2. In wild-type cells, Sip1 was less abundant than Sip2; nevertheless, the relative contribution of Sip1 to ER stress response was greater than that of Sip2. Therefore, it is likely that, in the case of ER stress response, Sip1 has a higher activity per molecule than Sip2. What is regulated in ER stress response by the β subunits? The β subunits are believed to function in determination of substrate preferences and subcellular localizations of the Snf1 complex^7^,^14^,^15^,^16^,^17^. However, these might not be the case for ER stress response, as their expression levels seem to be a critical determinant. Therefore, it should be further elucidated how the expression levels of three β subunits control the function of the Snf1 complex in ER stress response.

**Figure 6:**
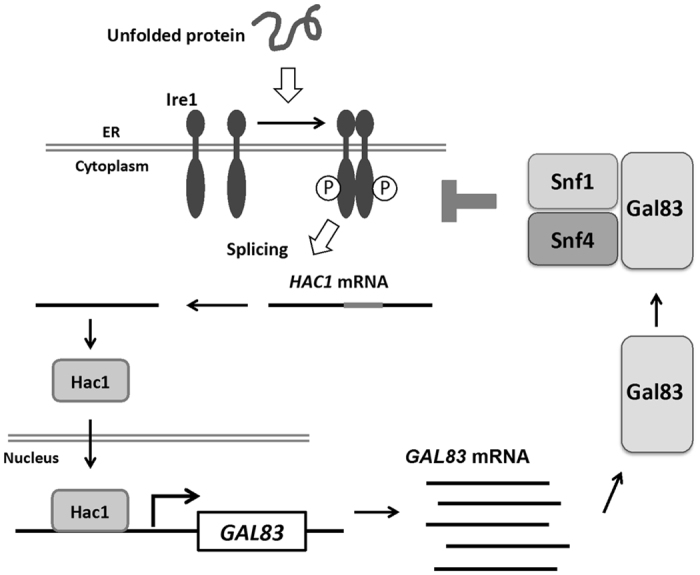
Proposed model for a feedback loop between the UPR and Snf1 in ER stress response.

Previous reports showed that a shift from fermentable to nonfermentable carbon sources upregulates the expression level of Sip2^15^. However, it remains unclear how Sip2 expression is modulated by carbon sources. A previous study using mammalian cells has revealed that the β1 subunit of AMPK is induced by cold stress and chemotherapeutic drug^30^; however, induction mechanism remains unclear. Therefore, it has yet to be elucidated how environmental changes alter the expression levels of the β subunits. In this study, we showed that transcription of the *GAL83* gene is activated rapidly and transiently by ER stress. In yeast ER stress response, several signaling pathways, including the UPR, Mpk1, Hog1, and Snf1, become activated^1^,^2^,^3^,^4^,^5^,^6^,^19^. ER stress induced-activation of Mpk1, Hog1, and Snf1 was maintained long-term. In contrast, the UPR activity was increased rapidly after ER stress treatment and gradually decreased thereafter. In accord with the rapid and transient activation of the UPR, expression of the well-known UPR target genes, such as *ERO1* and *KAR2*, was induced rapidly and transiently following exposure to ER stress^19^,^31^. Similar expression pattern was seen in the *GAL83* gene. Furthermore, induction of *GAL83* was impaired in *hac1* and *ire1* mutant cells. These observations suggest that the *GAL83* gene is directly controlled by the UPR during ER stress response. Consistent with our previous finding that Snf1 negatively regulates the UPR^19^, we found here that the expression level of *GAL83* was downregulated in Snf1-hypreactivated cells. This suggests that Snf1 negatively regulates itself through transcriptional inhibition of its regulatory subunit Gal83. On the other hand, it is also possible that the UPR negatively regulates itself through potentiating Snf1 function, based on the observation that Gal83 expression was induced in a manner dependent on the UPR. Taken together, the UPR and Snf1 may form a feedback loop to modulate the signal mediating ER stress response (Fig. 6). Since failure of the UPR to be downregulated properly results in hypersensitivity to ER stress^31^,^32^, it may be anticipated that the defect in Snf1-mediated feedback inhibition of the UPR causes ER stress sensitive phenotype. However, cells deleted for components of the Snf1 complex did in fact display resistance to ER stress. Why does loss of Snf1 function cause ER stress resistance phenotype? We have previously shown that in *snf1* mutant cells, the basal activity of the UPR is increased compared with wild-type cells, but attenuation of the UPR activity is apparently normal^19^. This observation suggests that upregulated, but controllable, UPR activity possibly contributes to ER stress resistant phenotype observed in the *snf1* mutants, and further indicates that Snf1 plays an auxiliary role in downregulation of the UPR. Previous studies also revealed that the changes of Ire1 phosphorylation state lead to attenuation of the UPR^31^,^32^. Therefore, it is possible that Snf1 participates in modulating the phosphorylation state of Ire1. Thus, further analyses should be needed to reveal the physiological importance of the feedback regulation between the UPR and Snf1, and will provide valuable insights into the mechanism to finely tune the UPR during ER stress response.

## Materials and Methods

### Strains

Strains used in this study are listed in Table 1 and Supplementary Table 1. Yeast strains harboring the complete gene deletions and carboxyl-terminally GFP-tagged genes were generated by a PCR-based method as described previously^33^. BY4741 and its mutant derivatives, *reg1, hac1*, and *ire1*, were obtained from Open Biosystems. All other strains were constructed by a PCR-based method and verified by PCR to confirm that replacement had occurred at the expected locus. Standard procedures were followed for yeast manipulations^34^.

### Plasmids

Plasmids used in this study are described in Table 2. In-Fusion cloning kits (Takara) was used to construct plasmids. The *P*_*GAL83*_*-GFP* and *P*_*SIP2*_*-GFP* were constructed as follows. The DNA fragment encoding GFP followed by the *ADH1* terminator (*T*_*ADH1*_) was obtained by PCR using the pFA6a-GFP vector^33^ as a template. The *GFP-T*_*ADH1*_ DNA fragment was fused to 698-bp and 690-bp genomic fragments containing 5′ upstream sequences of the *GAL83* and *SIP2* genes, respectively, yielding the *P*_*GAL83*_*-GFP* and *P*_*SIP2*_*-GFP* plasmids. The *P*_*GAL83*_*-SIP2* was constructed as follows. The coding region of the *SIP2* gene together with a 537-bp 3′ downstream sequence was amplified by PCR using genomic DNA as a template. The *SIP2* DNA fragment was fused to a 698-bp genomic fragment containing 5′ upstream sequences of the *GAL83* gene, yielding the *P*_*GAL83*_*-SIP2* plasmid. Schemes detailing construction of plasmids and primer sequences are available on request.

### Protein extraction, western blot analysis and antibodies

Preparation of protein extracts and Western blot analysis were performed as described previously^19^. Anti-GFP monoclonal antibody JL-8 (Clontech), anti-phospho-p38 MAPK monoclonal antibody D3F9 (Cell Signaling), anti-Hog1 polyclonal antibody y-215 (Santa Cruz), anti-phospho-p44/42 MAPK polyclonal antibody (Cell Signaling), anti-Mpk1 polyclonal antibody yN-19 (Santa Cruz), and anti-Mcm2 polyclonal antibody N-19 (Santa Cruz) were used. Detection was carried out by using a LAS-4000 (Fuji Film) with Immobilon Westren (Merck Millipore). Signal intensities were quantified by ImageQuant (GE Healthcare), and statistical analysis was performed with Excel (Microsoft).

### RNA isolation and RT–PCR

Preparation of total RNA and generation of cDNA were performed as described previously^19^. The *HAC1* cDNA was amplified from first strands of cDNA with Blend Taq (TOYOBO), and then analyzed by agarose gel electrophoresis. Detection, quantification, and statistical analysis was carried out by using a LAS-4000 (Fuji Film), ImageQuant (GE Healthcare), and Excel (Microsoft), respectively. The cDNA of *GFP* was quantitated by a quantitative real-time RT-PCR (qRT-PCR) method using a 7500 fast real-time RT-PCR system (Applied Biosystems) with SYBR Premix Ex Taq (Takara). A standard curve was generated from diluted RNA derived from wild-type cells, and levels of gene expression were normalized to *ACT1* expression. *HAC1* primers (CTGGCTGACCACGAAGACGC and TTGTCTTCATGAAGTGATGA) were used to monitor splicing of *HAC1* mRNA. *GAL83* primers (CAGCTGCCTCCAGGTACTCA and GGTCGGTTGCGGTAGGTAAA), *SIP1* primers (CAGTCCTTCTACTCAGGATCCATCG and TGAGAGGTTATGCTTCCCTGACG), *SIP2* primers (CCAGCGATCGATCCTCAATTGC and ACGGCGGGAATGTCTGTTGTATA), *GFP* primers (GGAGAGGGTGAAGGTGATGC and CTTCGGGCATGGCACTCTTG), and *ACT1* primers (TGCCGAAAGAATGCAAAAGG and TCTGGAGGAGCAATGATCTTGA) were used to analyze the mRNA level.

### Stress sensitivity

Assays for tunicamycin toxicity were carried out as follows. Cells were grown to exponential phase, and cultures were adjusted to an optical density of 0.5. Cell cultures were then serially diluted 5-fold, spotted onto normal plates or plates containing the indicated concentrations of tunicamycin, followed by incubation at 25 °C for 3 days (for plates lacking tunicamycin) or more than 5 days (for plates containing tunicamycin).

## Additional Information

**How to cite this article:** Kimura, Y. *et al*. Expression control of the AMPK regulatory subunit and its functional significance in yeast ER stress response. *Sci. Rep.* **7**, 46713; doi: 10.1038/srep46713 (2017).

**Publisher's note:** Springer Nature remains neutral with regard to jurisdictional claims in published maps and institutional affiliations.

## Supplementary Material

Supplementary Information

## Figures and Tables

**Table 1:** Strains used in this study.

Strain	Genotype	Source
10BD	*MAT***a***/MAT***α** *ade2/ade2 trp1/trp1 can1/can1 leu2/leu2 his3/his3 ura3/ura3*	36
10B	*ade2 trp1 can1 leu2 his3 ura3 GAL psi* + *HOp-ADE2-HO 3*′ *UTR*	36
YSC1	*ade2 trp1 can1 leu2 his3 ura3 reg1::CgLEU2*	19
YSC2	*ade2 trp1 can1 leu2 his3 ura3 reg1::CgLEU2 sip1::kanMX6*	this study
YSC3	*ade2 trp1 can1 leu2 his3 ura3 reg1::CgLEU2 sip2::CgHIS3*	this study
YSC4	*ade2 trp1 can1 leu2 his3 ura3 reg1::CgLEU2 gal83::CgTRP1*	this study
YSC5	*ade2 trp1 can1 leu2 his3 ura3 reg1::LEU2 sip1::kanMX6 sip2::CgHIS3*	this study
YSC6	*ade2 trp1 can1 leu2 his3 ura3 reg1::LEU2 sip1::kanMX6 gal83::CgTRP1*	this study
YSC7	*ade2 trp1 can1 leu2 his3 ura3 reg1::LEU2 sip2::CgHIS3 gal83::CgTRP1*	this study
YSC8	*ade2 trp1 can1 leu2 his3 ura3 reg1::LEU2 sip1::kanMX6 sip2::CgHIS3 gal83::CgTRP1*	this study
YSC9	*ade2 trp1 can1 leu2 his3 ura3 SIP1-GFP::HIS3MX6*	this study
YSC10	*ade2 trp1 can1 leu2 his3 ura3 SIP2-GFP::HIS3MX6*	this study
YSC11	*ade2 trp1 can1 leu2 his3 ura3 GAL83-GFP::HIS3MX6*	this study
YSC12	*ade2 trp1 can1 leu2 his3 URA3::P*_*SIP2*_*-GFP*	this study
YSC13	*ade2 trp1 can1 leu2 his3 URA3::P*_*GAL83*_*-GFP*	this study
YSC14	*ade2 trp1 can1 leu2 his3 URA3::SIP2 reg1::LEU2 sip1::kanMX6 sip2::CgHIS3 gal83::CgTRP1*	this study
YSC15	*ade2 trp1 can1 leu2 his3 URA3::GAL83 reg1::LEU2 sip1::kanMX6 sip2::CgHIS3 gal83::CgTRP1*	this study
YSC16	*ade2 trp1 can1 leu2 his3 URA3::PGAL83-SIP2 reg1::LEU2 sip1::kanMX6 sip2::CgHIS3 gal83::CgTRP1*	this study
YSC18	*ade2 trp1 can1 leu2 his3 ura3 hac1::kanMX6 GAL83-GFP::HIS3MX6*	this study
YSC19	*ade2 trp1 can1 leu2 his3 ura3 ire1::kanMX6 GAL83-GFP::HIS3MX6*	this study
YSC20	*ade2 trp1 can1 leu2 his3 ura3 hac1::kanMX6*	19
YSC21	*ade2 trp1 can1 leu2 his3 ura3 ire1::kanMX6*	this study
YSC22	*ade2 trp1 can1 leu2 his3 ura3 snf1::CgTRP1*	19
YSC23	*ade2 trp1 can1 leu2 his3 ura3 reg1::CgLEU2 snf1::CgTRP1*	19
YSC24	*ade2 trp1 can1 leu2 his3 ura3 sip1::kanMX6 sip2::CgHIS3*	this study
YSC25	*ade2 trp1 can1 leu2 his3 ura3 sip1::kanMX6 gal83::KlURA3*	this study
YSC26	*ade2 trp1 can1 leu2 his3 ura3 sip2::CgHIS3 gal83::KlURA3*	this study

All strains were W303 derivatives.

**Table 2:** Plasmids used in this study.

Plasmids	Relevant markers	Source
pCgLEU2	*C. glabrata LEU2* in pUC19	37
pCgHIS3	*C. glabrata HIS3* in pUC19	37
pCgTRP1	*C. glabrata TRP1* in pUC19	37
pKlURA3	*K. lactis URA3* in pUC19	37
pFA6a-kanMX6	*kanMX6*	33
pFA6a-GFP-HIS3MX6	*GFP-ADH 3*′*UTR-HIS3MX6*	33
pRS306	*URA3*	38
pRS306-P_SIP2_-GFP	*URA3, P*_*SIP2*_*-GFP*	this study
pRS306-P_GAL83_-GFP	*URA3, P*_*GAL83*_*-GFP*	this study
pRS306-SIP2	*URA3, SIP2*	this study
pRS306-GAL83	*URA3, GAL83*	this study
pRS306-P_GAL83_-SIP2	*URA3, P*_*GAL83*_*-SIP2*	this study
