# Supplementary material for: Expression control of the AMPK regulatory subunit and its functional significance in yeast ER stress response
Source: Sci Rep. 2017 Apr 21;7:46713. doi: 10.1038/srep46713 (PMC5399461; doi:10.1038/srep46713)
Supplement: Supplementary Information [file srep46713-s1.pdf]

Title:

Expression control of the AMPK regulatory subunit and its functional significance in yeast ER stress response

Authors:

Yuichi Kimura, Kenji Irie and Tomoaki Mizuno*

Affiliation:

Department of Molecular Cell Biology, Faculty of Medicine, University of Tsukuba, Tsukuba, Japan.

*Correspondence and requests for materials should be addressed to T. M. (mizuno@md.tsukuba.ac.jp)

Supplementary Figure 1

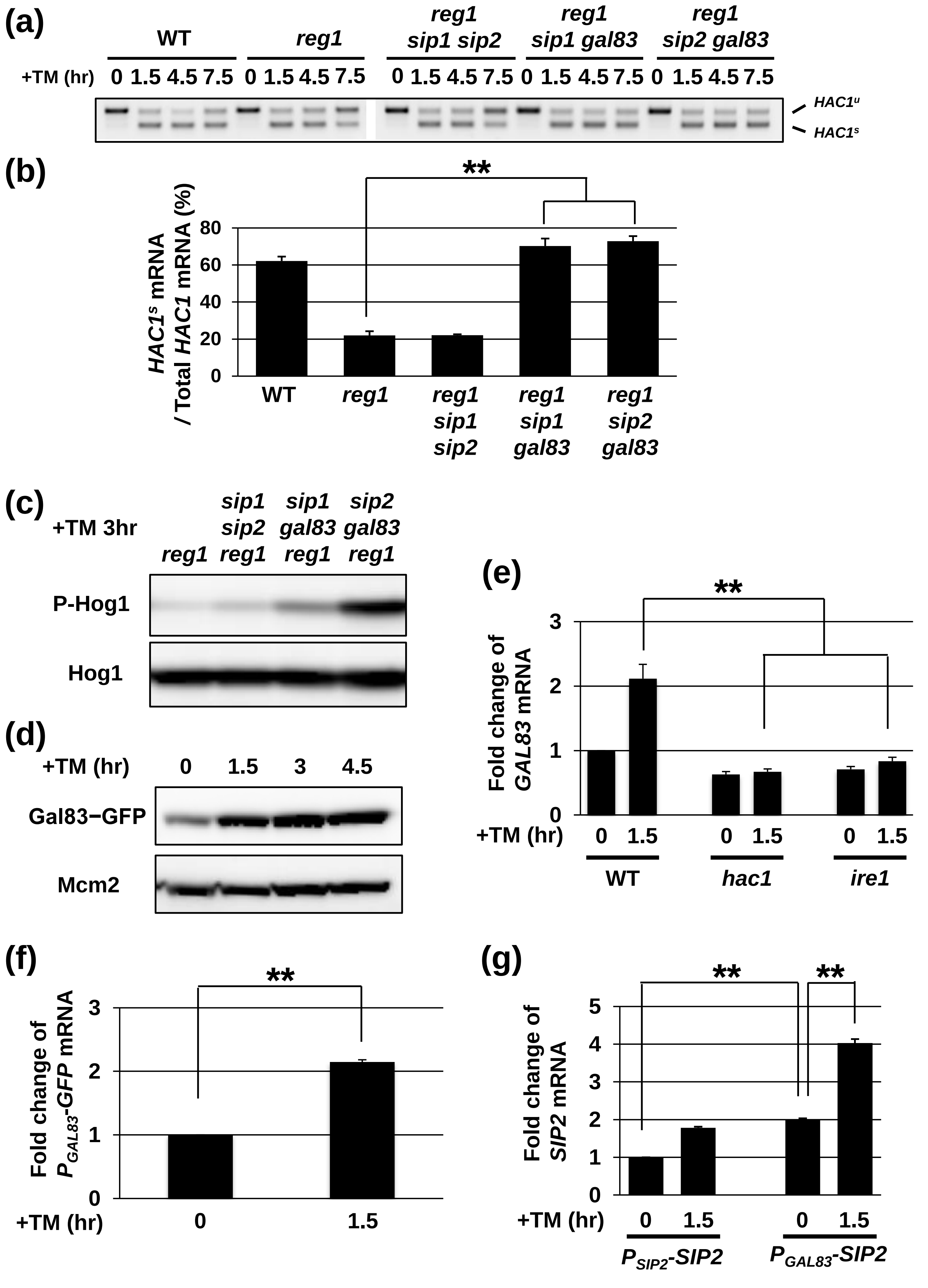

Supplementary Fig. 1. The levels of the UPR and Hog1 activation and *GAL83* expression after tunicamycin treatment.

(a, b) Splicing of *HAC1* mRNA after tunicamycin treatment. Wild-type (WT) and indicated mutant strains were treated with 0.4 μg/ml tunicamycin (TM) for the indicated time. Total RNAs prepared from each strain were subjected to RT-PCR of *HAC1*. Positions of unspliced *HAC1* ($HAC1^u$) and spliced *HAC1* ($HAC1^s$) are indicated. The mean of $HAC1^s/(HAC1^u + HAC1^s)$ at 7.5 hours after tunicamycin addition with SEM (n = 3) is shown in (B). $^{**}P < 0.01$ as determined by Tukey's test.

(c) Hog1 activation after tunicamycin treatment. Indicated mutant strains were treated with 2 μg/ml tunicamycin (TM) for 3 hours. Extracts prepared from each cell were immunoblotted with anti-phospho-p38 (P-Hog1) and anti-Hog1 antibodies.

(d) The expression levels of Gal83 after tunicamycin treatment. The strain harboring GFP-tagged *GAL83* were treated with 2 μg/ml tunicamycin (TM) for the indicated time. Extracts prepared from each cell were immunoblotted with anti-GFP and anti-Mcm2 antibodies.

(e) The mRNA levels of *GAL83* after tunicamycin treatment. Wild-type (WT) and indicated mutant strains were treated with 2 μg/ml tunicamycin (TM) for the indicated time. The mRNA levels were quantified by qRT-PCR analysis, and relative mRNA levels were calculated using *ACT1* mRNA. The data show mean ± SEM (n = 4). $^{**}P < 0.01$ as determined by Tukey's test.

(f) Effects of tunicamycin treatment on expression of P_{GAL83}-*GFP* reporter. Wild-type (WT) cells harboring the integration which expresses GFP under the control of *GAL83* promoter were treated with 2 μg/ml tunicamycin (TM) for the indicated time. The *GFP* mRNA levels were quantified by qRT-PCR analysis, and relative mRNA levels were calculated using *ACT1* mRNA. The data show mean ± SEM (n = 4). $^{*}P < 0.05$ as determined by Student's *t*-test.

(g) Comparison between *SIP2* expression levels from *SIP2* and P_{GAL83}-*SIP2*. The *reg1 sip1 sip2 gal83* quadruple mutant strains harboring the integration of P_{SIP2}-*SIP2* or P_{GAL83}-*SIP2* were treated with 2 μg/ml tunicamycin (TM) for the indicated time. The *SIP2* mRNA levels were quantified by qRT-PCR analysis, and relative mRNA levels were calculated using *ACT1* mRNA. The data show mean ± SEM (n = 3). $^{*}P < 0.05$ as determined by Tukey's test.

Supplementary Figure 2

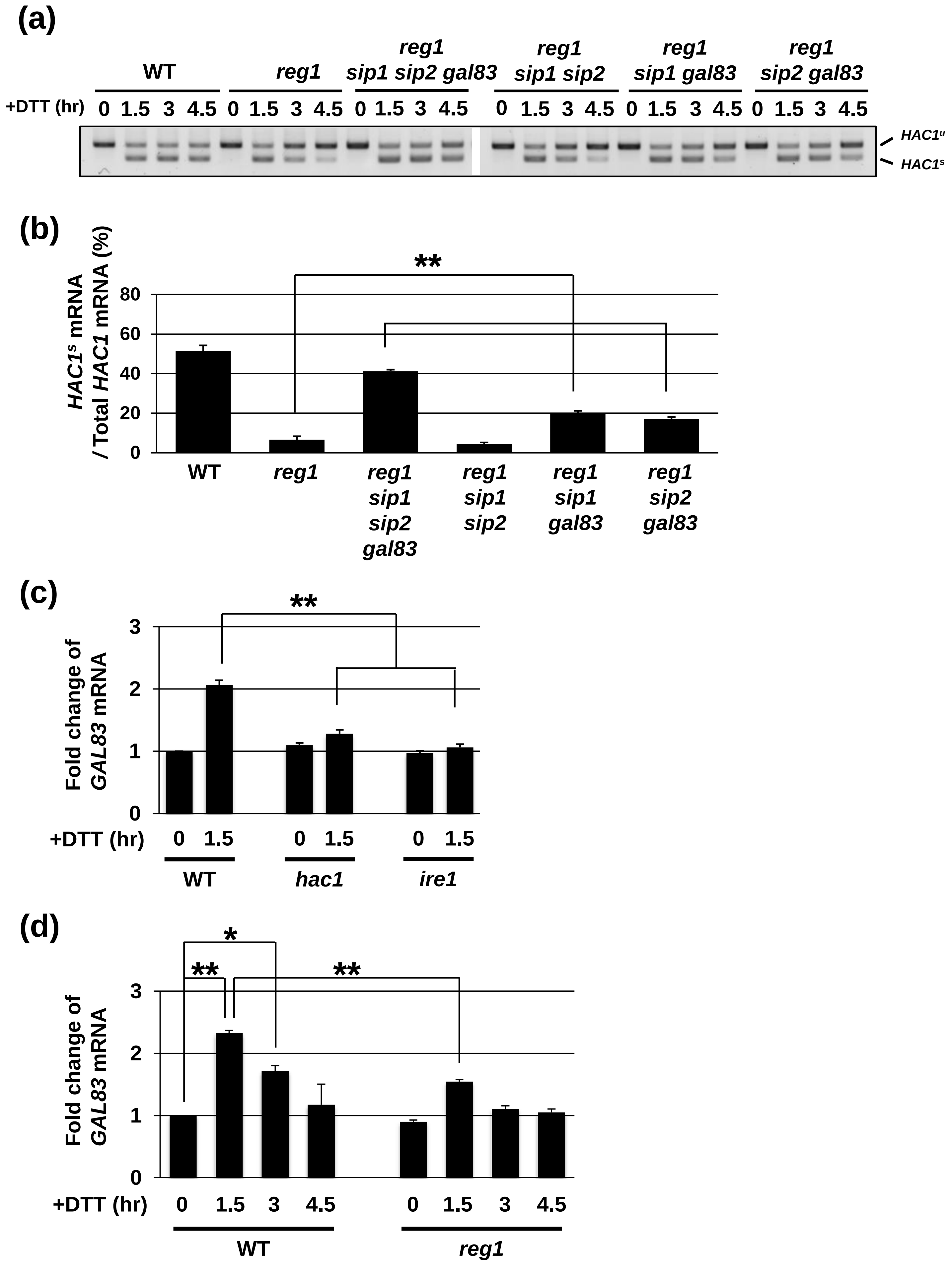

Supplementary Fig. 2. Regulation of the UPR activity and *GAL83* expression in the BY4741 derivatives.

(a, b) Splicing of *HAC1* mRNA in the BY4741 background strains. Wild-type (WT) and indicated mutant strains were treated with 4 mM dithiothreitol (DTT) for the indicated time. Total RNAs prepared from each strain were subjected to RT-PCR of *HAC1*. Positions of unspliced *HAC1* ($HAC1^u$) and spliced *HAC1* ($HAC1^s$) are indicated. The mean of $HAC1^s/(HAC1^u + HAC1^s)$ at 4.5 hours after DTT addition with SEM (n = 4) is shown in (B). $^{**}P < 0.01$ as determined by Tukey's test.

(c) The mRNA levels of *GAL83* in the BY4741 background strains. Wild-type (WT) and indicated mutant strains were treated with 4 mM dithiothreitol (DTT) for the indicated time. The mRNA levels were quantified by qRT-PCR analysis, and relative mRNA levels were calculated using *ACT1* mRNA. The data show mean ± SEM (n = 4). $^{**}P < 0.01$ as determined by Tukey's test.

(d) Effects of the *reg1* mutation on ER stress-induced upregulation of *GAL83* mRNA in the BY4741 background strains. Wild-type (WT) and *reg1* mutant strains were grown at 25 ° C until exponential phase and treated with 4 mM dithiothreitol (DTT) for the indicated time. The mRNA levels were quantified by qRT-PCR analysis, and relative mRNA levels were calculated using *ACT1* mRNA. The data show mean ± SEM (n = 4). $^{*}P < 0.05$ and $^{**}P < 0.01$ as determined by Tukey's test.

Supplementary Figure 3

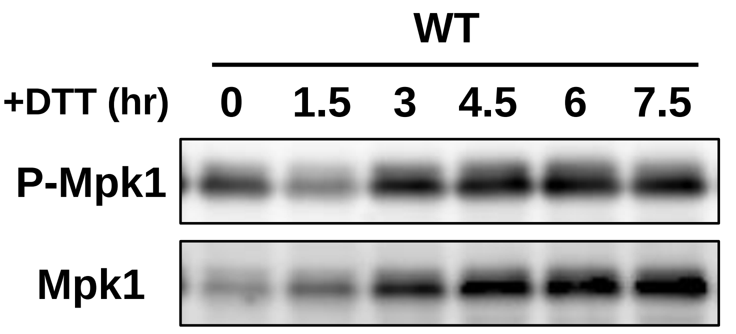

Supplementary Fig. 3. The Mpk1 activity during ER stress response.
Wild-type (WT) cells were treated with 4 mM dithiothreitol (DTT) for the indicated time. Extracts prepared from each cell were immunoblotted with anti-phospho-p44/42 (P-Mpk1) and anti-Mpk1 antibodies.

Supplementary Figure 4

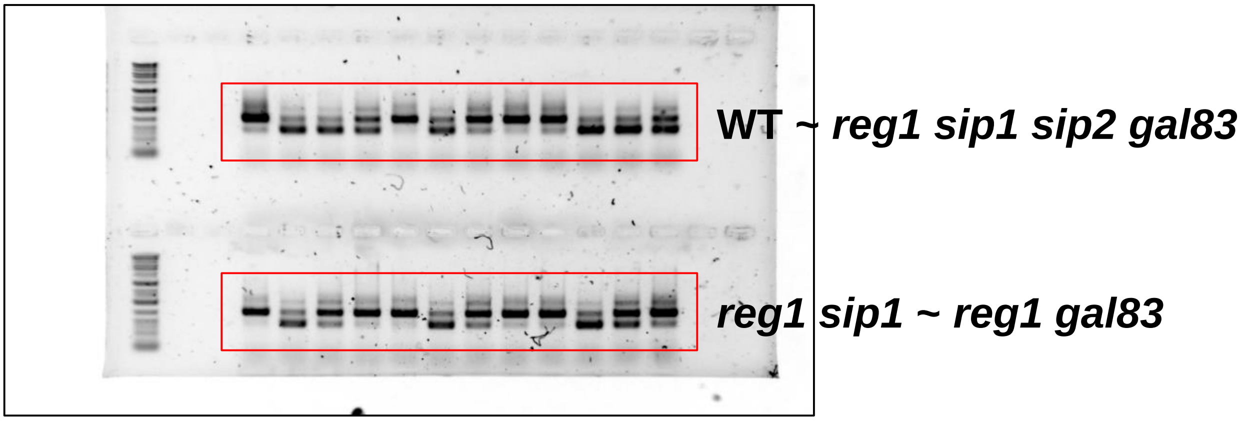

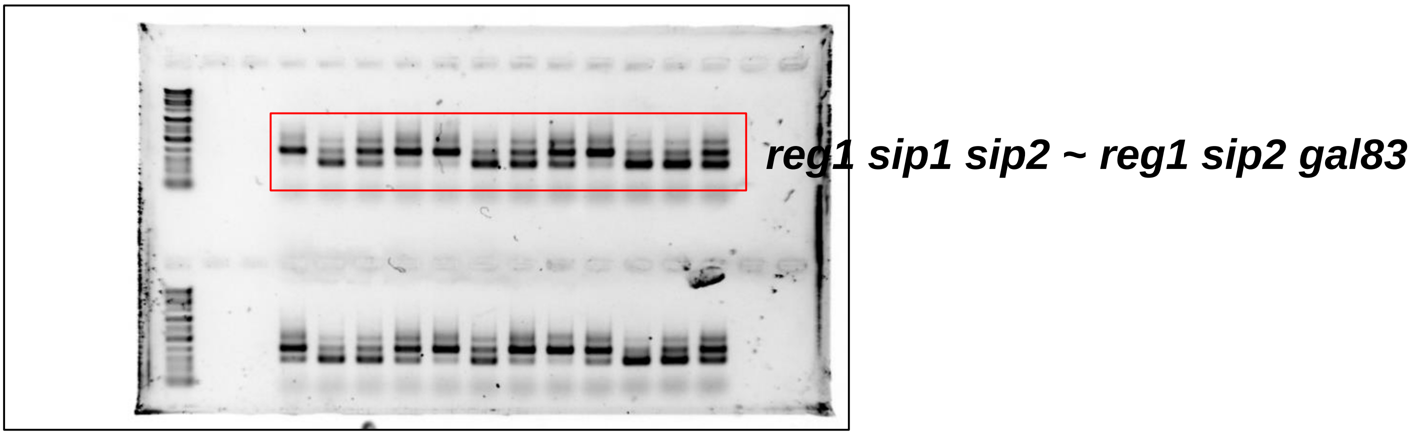

Supplementary Fig. 4. Original data for Fig. 1a.

Supplementary Figure 5

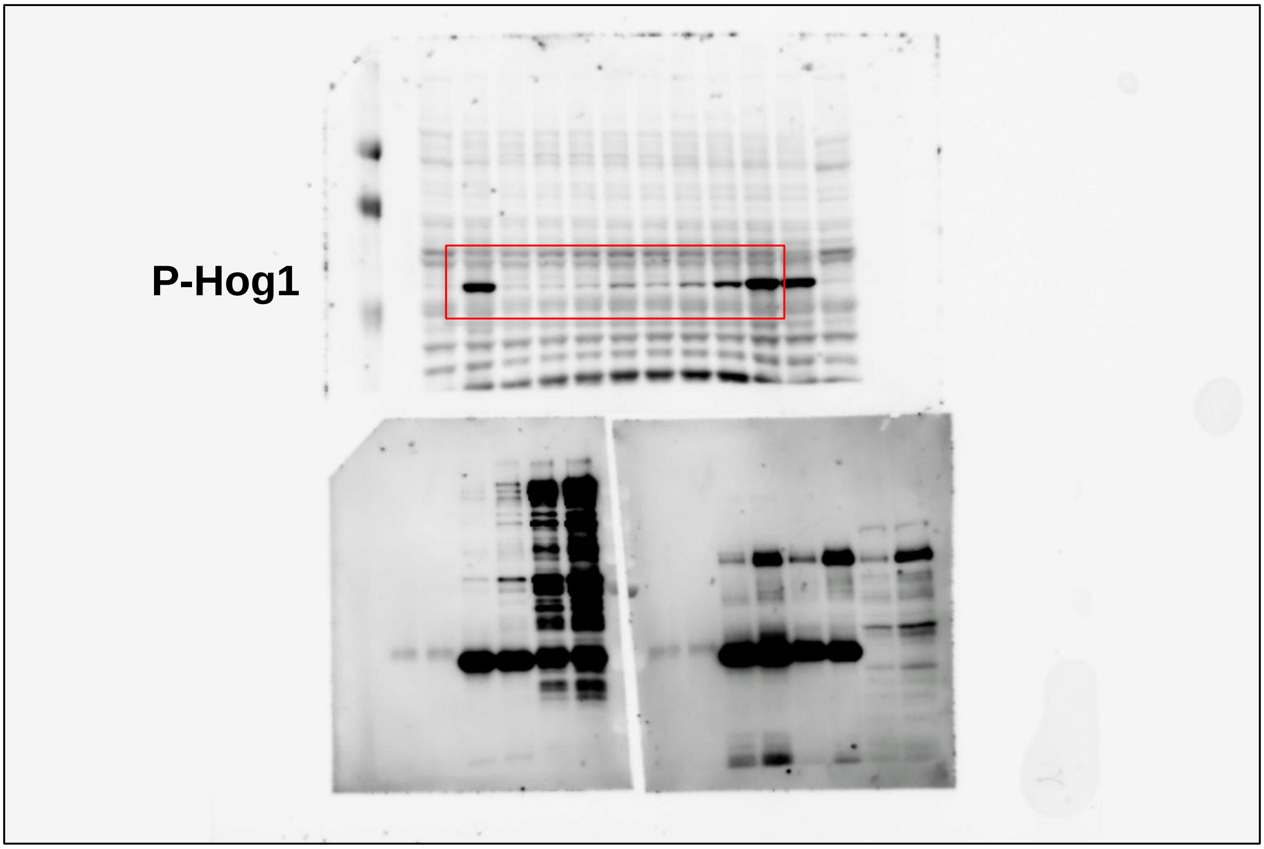

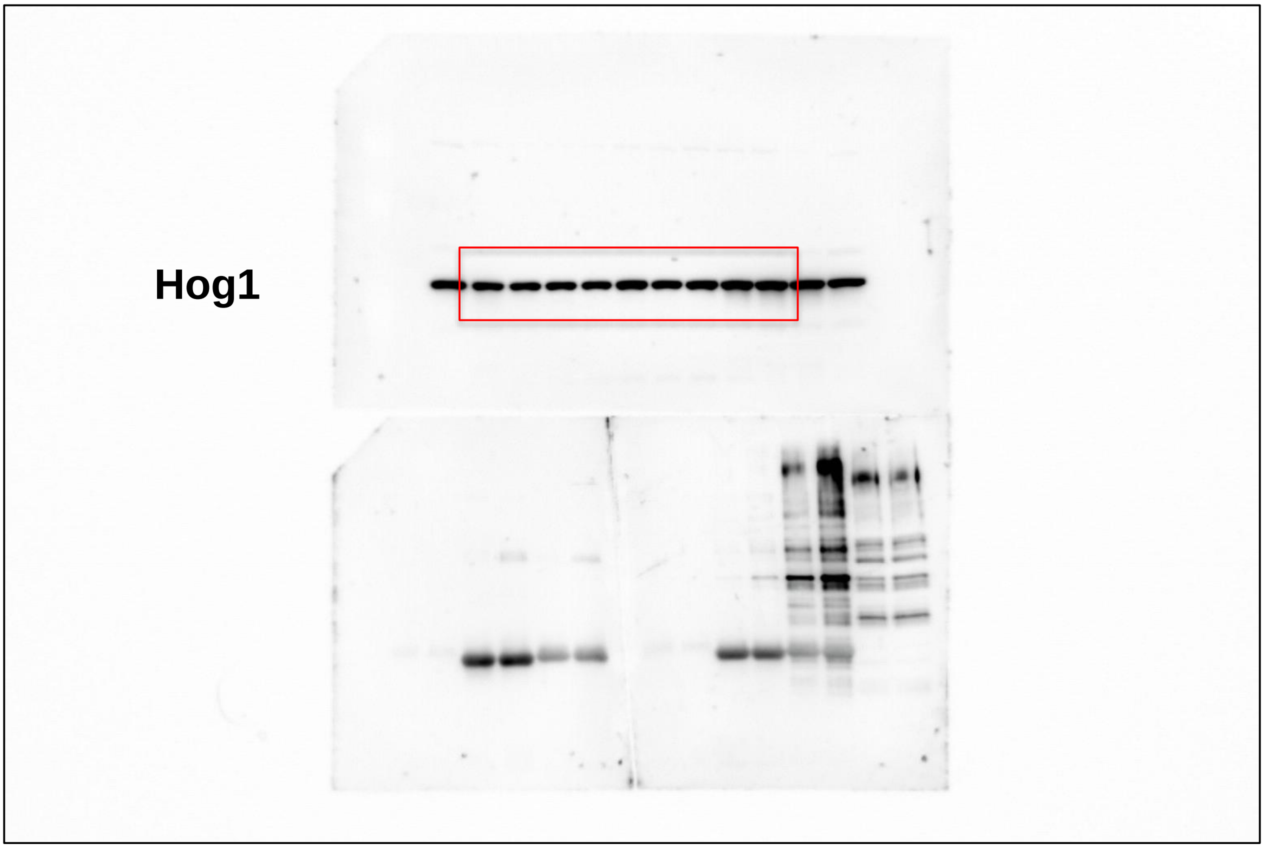

Supplementary Fig. 5. Original data for Fig. 1c.

Supplementary Figure 6

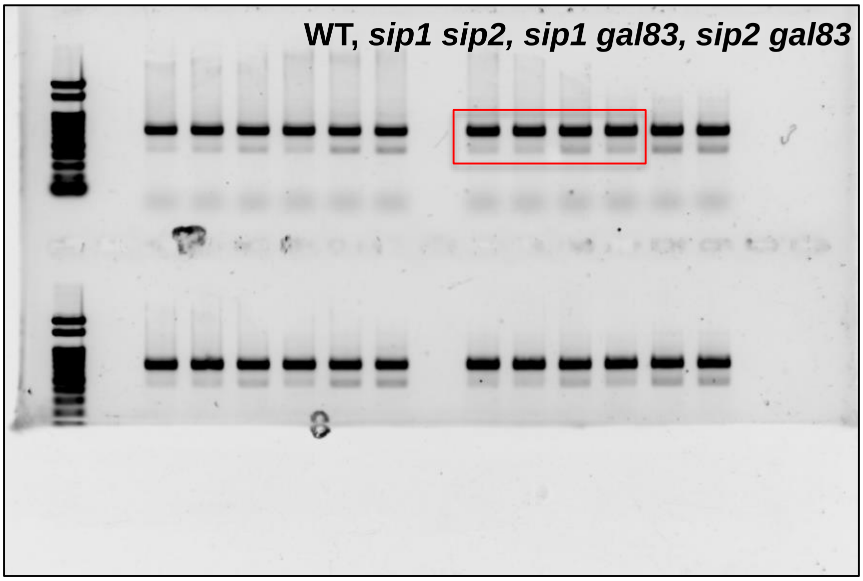

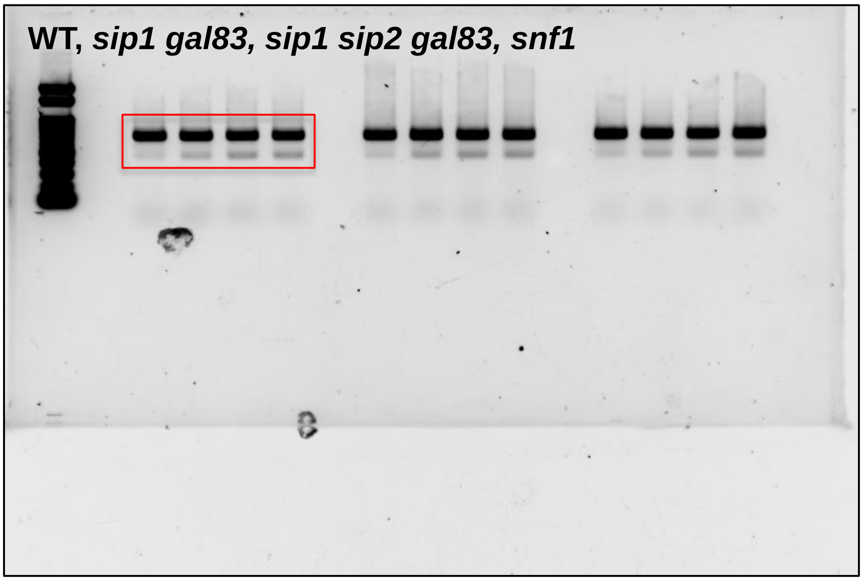

Supplementary Fig. 6. Original data for Fig. 2a.

Supplementary Figure 7

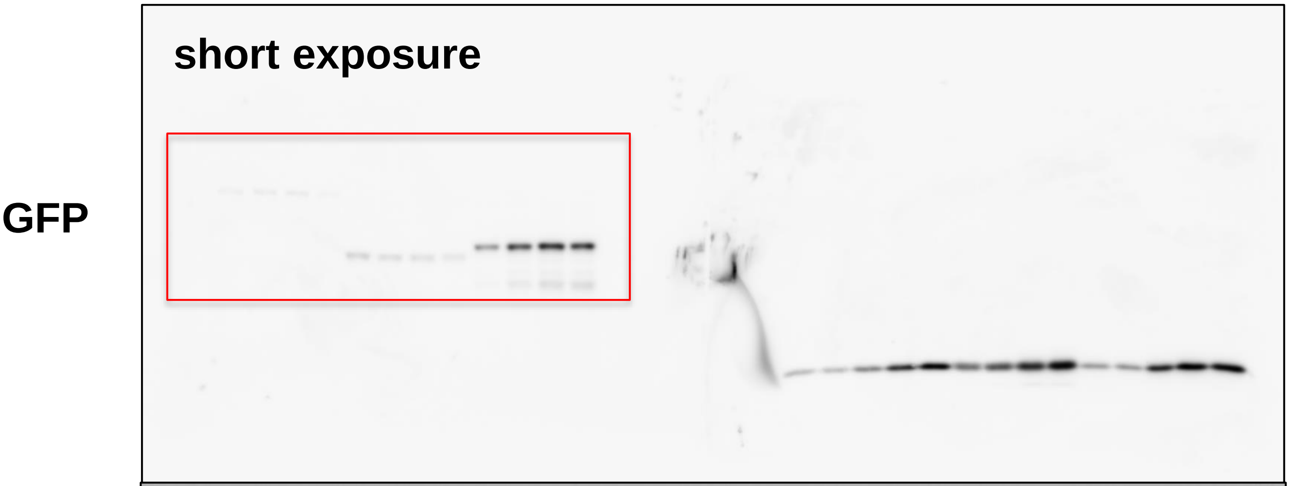

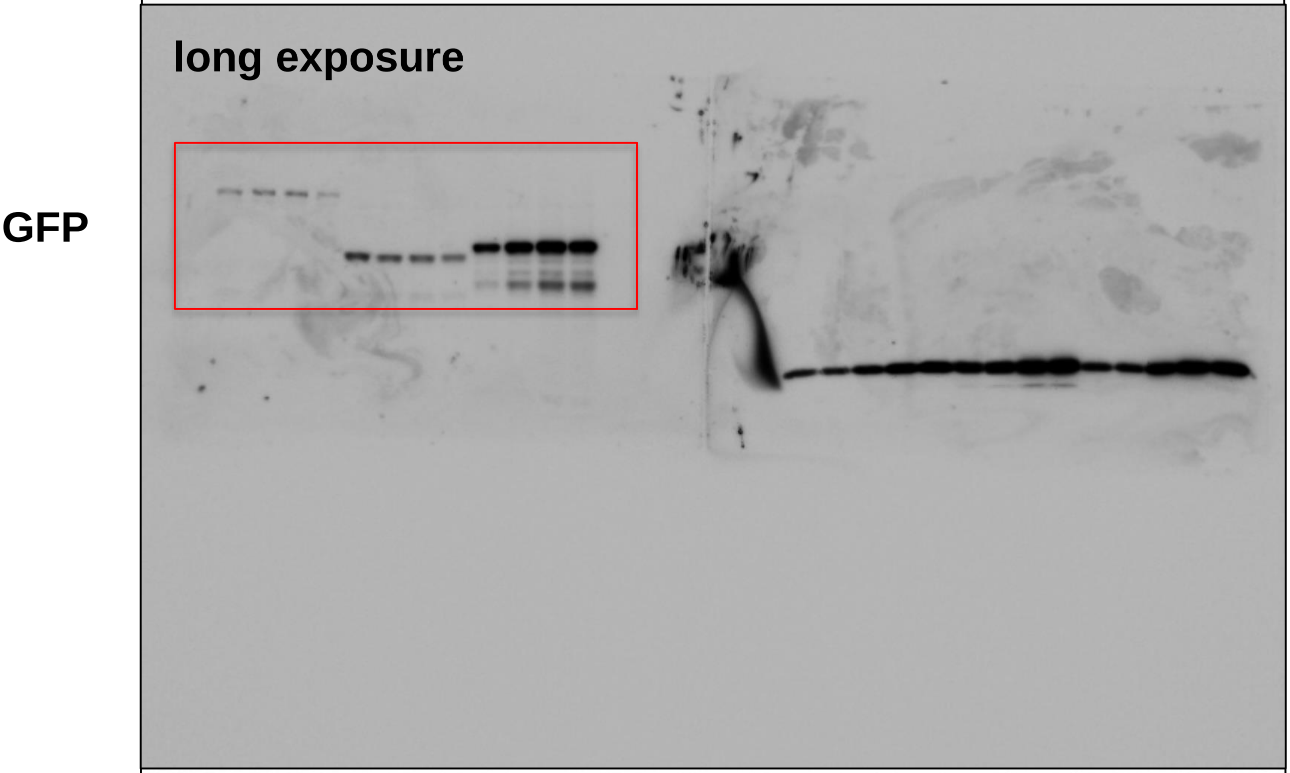

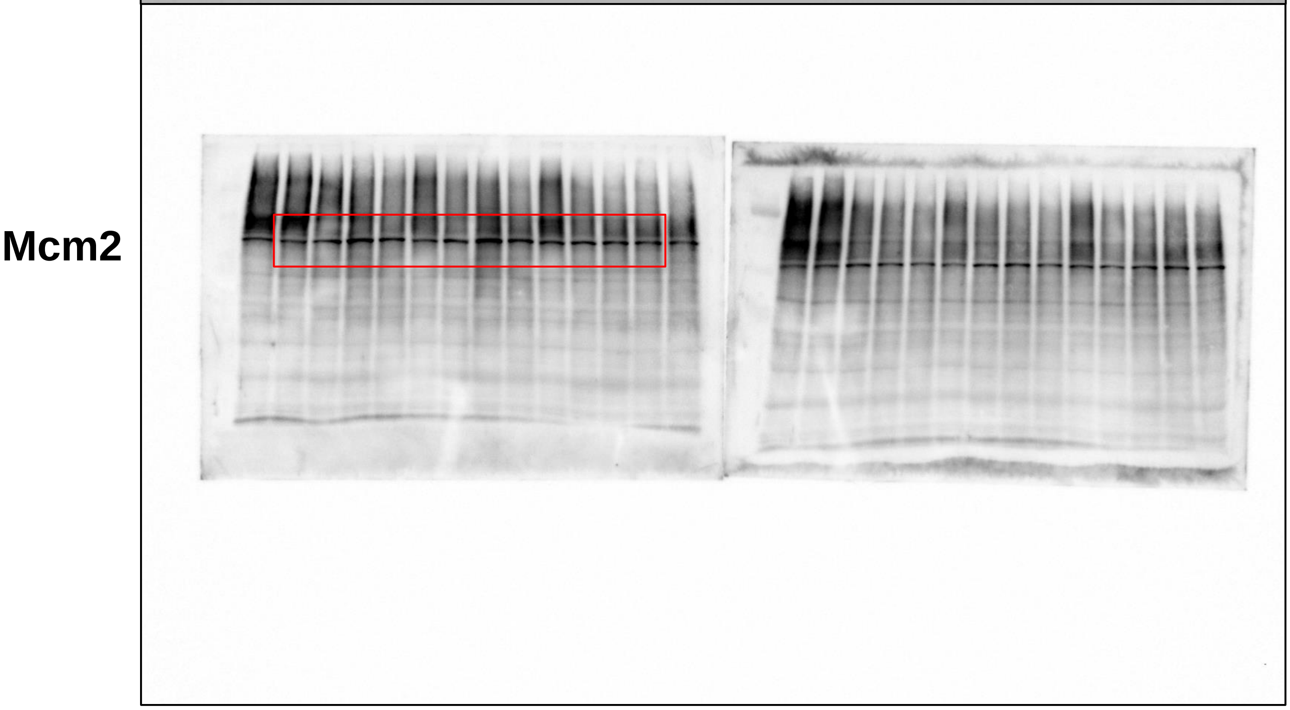

Supplementary Fig. 7. Original data for Fig. 3a.

Supplementary Figure 8

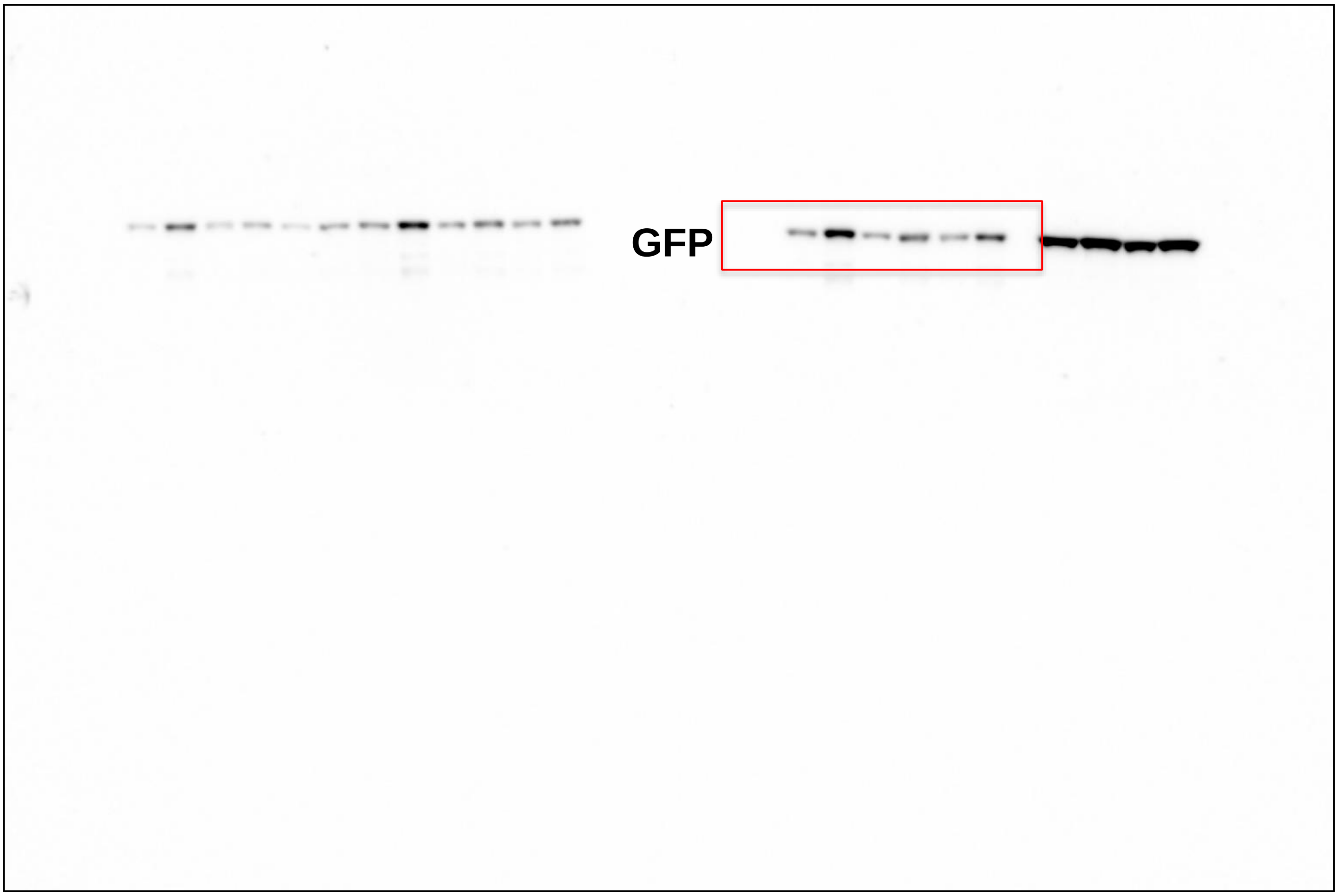

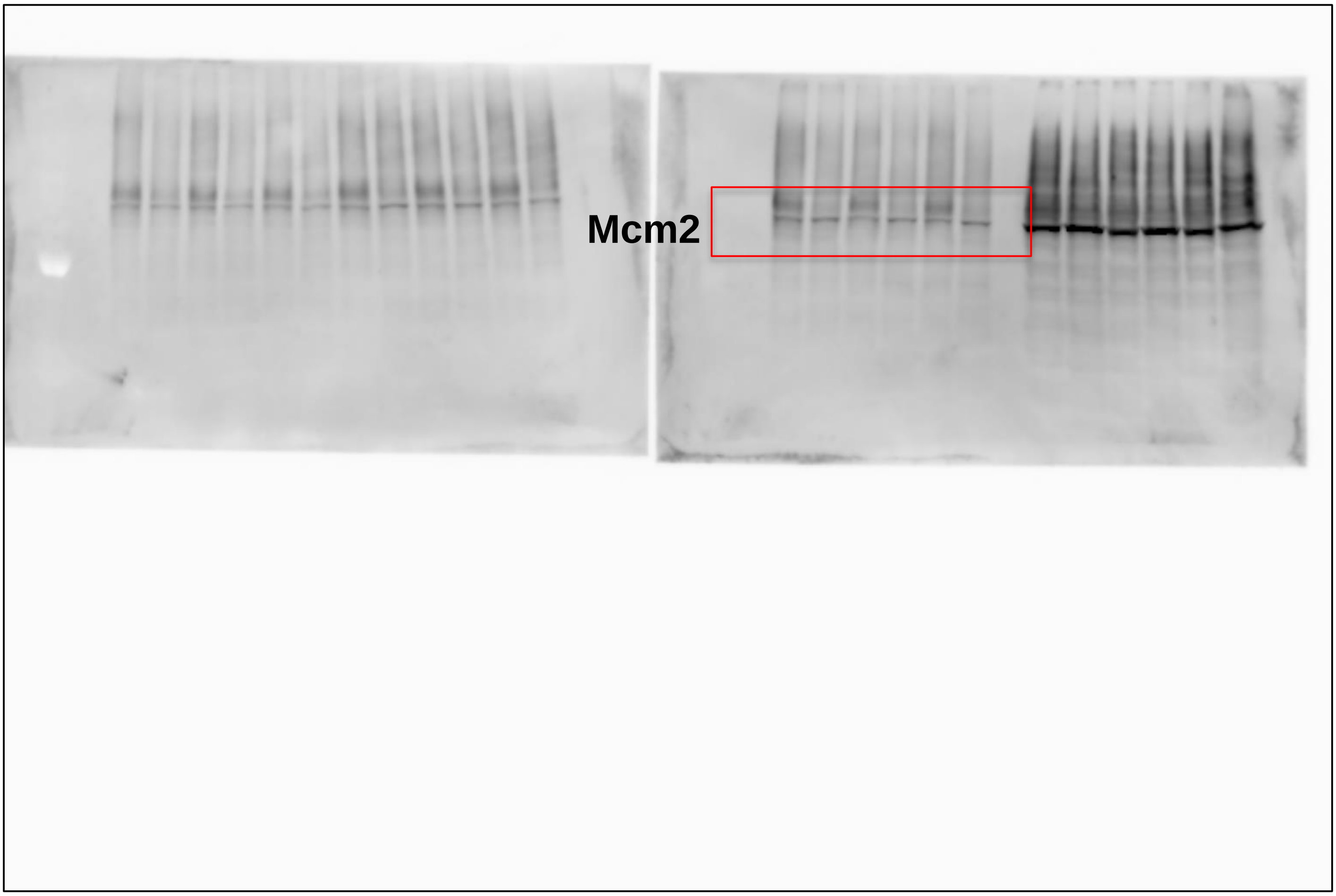

Supplementary Fig. 8. Original data for Fig. 4a.

Supplementary Table 1

Strain	Genotype	Source
BY4741	*MAT* **a** *met15 leu2 his3 ura3*	39
3967	*MAT* **a** *met15 leu2 his3 ura3 reg1::kanMX*	39
5650	*MAT* **a** *met15 leu2 his3 ura3 hac1::kanMX*	39
1907	*MAT* **a** *met15 leu2 his3 ura3 ire1::kanMX*	39
YSC27	*MAT* **a** *met15 leu2 his3 ura3 reg1::kanMX sip1::CgLEU2 sip2::CgHIS3*	this study
YSC28	*MAT* **a** *met15 leu2 his3 ura3 reg1::kanMX sip1::CgLEU2 gal83::KlURA3*	this study
YSC29	*MAT* **a** *met15 leu2 his3 ura3 reg1::kanMX sip2::CgHIS3 gal83::KlURA3*	this study
YSC30	*MAT* **a** *met15 leu2 his3 ura3 reg1::kanMX sip1::CgLEU2 sip2::CgHIS3 gal83::KlURA3*	this study

All strains were BY4741 derivatives.

39. Brachmann, C. B., Davies, A., Cost, G. J., Caputo, E., Li, J., Hieter, P. & Boeke, J. D. Designer deletion strains derived from *Saccharomyces cerevisiae* S288C: a useful set of strains and plasmids for PCR-mediated gene disruption and other applications. *Yeast* **14**, 115-132 (1998).
